# Dynamic Deformation Analysis of Super High-Rise Buildings Based on GNSS and Accelerometer Fusion

**DOI:** 10.3390/s25092659

**Published:** 2025-04-23

**Authors:** Xingxing Xiao, Houzeng Han, Jian Wang, Dong Li, Cai Chen, Lei Wang

**Affiliations:** 1 School of Geomatics and Urban Spatial Informatics, Beijing University of Civil Engineering and Architecture, Beijing 102616, China; 1108130023033@stu.bucea.edu.cn (X.X.); wangjian@bucea.edu.cn (J.W.); 1108130023034@stu.bucea.edu.cn (D.L.); 1108130422001@stu.bucea.edu.cn (C.C.); 1108130023031@stu.bucea.edu.cn (L.W.); 2 Research Center for Urban Big Data Applications, Beijing University of Civil Engineering and Architecture, Beijing 100044, China

**Keywords:** dynamic deformation monitoring of super-tall buildings, global navigation satellite system (GNSS), adaptive robust Kalman filtering (ARKF), feature modal decomposition (FMD), Newton–Raphson optimization (NRBO)

## Abstract

To accurately capture the dynamic displacement of super-tall buildings under complex conditions, this study proposes a data fusion algorithm that integrates NRBO-FMD optimization with Adaptive Robust Kalman Filtering (ARKF). The NRBO-FMD method preprocesses GNSS and accelerometer data to mitigate GNSS multipath effects, unmodeled errors, and high-frequency noise in accelerometer signals. Subsequently, ARKF fuses the preprocessed data to achieve high-precision displacement reconstruction. Numerical simulations under varying noise conditions validated the algorithm’s accuracy. Field experiments conducted on the Hairong Square Building in Changchun further demonstrated its effectiveness in estimating three-dimensional dynamic displacement. Key findings are as follows: (1) The NRBO-FMD algorithm significantly reduced noise while preserving essential signal characteristics. For GNSS data, the root mean square error (RMSE) was reduced to 0.7 mm for the 100 s dataset and 1.0 mm for the 200 s dataset, with corresponding signal-to-noise ratio (SNR) improvements of 3.0 dB and 6.0 dB. For accelerometer data, the RMSE was reduced to 3.0 mm (100 s) and 6.2 mm (200 s), with a 4.1 dB SNR gain. (2) The NRBO-FMD–ARKF fusion algorithm achieved high accuracy, with RMSE values of 0.7 mm (100 s) and 1.9 mm (200 s). Consistent PESD and POSD values demonstrated the algorithm’s long-term stability and effective suppression of irregular errors. (3) The algorithm successfully fused 1 Hz GNSS data with 100 Hz accelerometer data, overcoming the limitations of single-sensor approaches. The fusion yielded an RMSE of 3.6 mm, PESD of 2.6 mm, and POSD of 4.8 mm, demonstrating both precision and robustness. Spectral analysis revealed key dynamic response frequencies ranging from 0.003 to 0.314 Hz, facilitating natural frequency identification, structural stiffness tracking, and early-stage performance assessment. This method shows potential for improving the integration of GNSS and accelerometer data in structural health monitoring. Future work will focus on real-time and predictive displacement estimation to enhance monitoring responsiveness and early-warning capabilities.

## 1. Introduction

The dynamic displacement of super-tall buildings is a critical parameter for evaluating their structural integrity and operational performance [1,2]. Compared to conventional buildings and bridges, monitoring the top displacement of super-tall structures poses considerable challenges [3]. Due to the height amplification effect, deformation measurement errors in complex environments are significantly magnified at the top of the structure, leading to reduced monitoring accuracy and cumulative error propagation [4,5]. Moreover, studies have shown that the dynamic displacement at the top of super-tall buildings often exhibits substantial high-frequency components, which are further superimposed with external environmental noise, thereby increasing measurement complexity and uncertainty [6,7,8]. These characteristics render the accurate monitoring of top displacement in super-tall buildings a critical yet challenging research focus [9].

In recent years, real-time dynamic displacement monitoring technologies have advanced rapidly [10]. Among them, the Global Navigation Satellite System (GNSS), featuring continuous, real-time, high-precision, and all-weather capabilities, has been widely employed in the deformation monitoring of super-tall buildings [11]. As early as the 1980s, the Canary Wharf Tower (244 m) in the United Kingdom became the first building to utilize satellite positioning technology for monitoring structural changes and displacement [12,13]. Since then, GNSS technology has also been applied to the deformation monitoring of The Shard, various skyscrapers in the London Financial District, and the London Olympic Tower [14]. During the monitoring process, GNSS enables the acquisition of three-dimensional displacement data, particularly suitable for capturing low-frequency structural deformations such as foundation settlement and wind-induced slow drift. However, GNSS typically operates at a low sampling rate (generally 1 Hz or lower). Although some commercial GNSS receivers are capable of reaching sampling rates up to 100 Hz, the accuracy of the solution significantly decreases as the sampling rate increases [15]. This degradation is primarily due to the amplification of internal receiver noise, multipath effects, and atmospheric interference at higher sampling rates, which can cause the signal to be overwhelmed by noise. Consequently, this limits the applicability of GNSS in high-frequency dynamic monitoring. In contrast, accelerometers possess a high-frequency response capability, enabling accurate measurement of a structure’s dynamic acceleration. High-frequency displacement information can then be derived through numerical integration. Accelerometers can reliably identify the modal parameters of a monitored structure and precisely detect high-frequency, low-amplitude motion with sub-centimeter accuracy [16].

At the same time, accelerometers, due to their high sampling rates, are widely used in structural health monitoring to extract vibration modes [17]. By integrating acceleration data, velocity and displacement in the monitoring direction can be derived, representing the dynamic deformation of structural components. However, cumulative errors during the integration process can lead to result divergence [18,19]. Extensive research has been conducted to address sensor noise and the accumulation of integration errors, and various improvement methods have been proposed [20]. For accelerometers, signal drift during double integration for displacement estimation significantly reduces the accuracy of long-term monitoring. Integration methods for accelerometer signals are generally classified into two categories: frequency-domain and time-domain approaches. Frequency-domain methods, such as those based on the Fast Fourier Transform (FFT), can effectively filter certain types of noise [21]. Experimental studies using vibration tables have demonstrated that the conversion of acceleration to displacement is susceptible to both high-frequency and low-frequency noise interference [22].

GNSS technologies such as Real-Time Kinematic (RTK) and Precise Point Positioning (PPP) have demonstrated excellent performance in dynamic displacement measurements. However, their signals are highly susceptible to multipath effects and satellite occlusion, which can significantly reduce data accuracy [23,24]. Consequently, their effectiveness in capturing long-term trends in structural deformation is limited. To enhance measurement accuracy, GNSS is often integrated with accelerometers in structural monitoring systems [25]. Despite this advancement, current multi-sensor fusion approaches still face challenges in handling complex noise and high-frequency signal components [26,27]. For example, the Kalman filter has limited ability to dynamically adapt to varying noise distributions, and sensor acquisition errors can lead to cumulative biases in the fusion process [28,29]. Moreover, the dynamic displacement responses at the tops of super-tall buildings under different monitoring conditions remain insufficiently investigated in existing research [30,31]. In the research on GNSS and accelerometer data fusion, the Multi-Rate Kalman Filter (MRKF) has become a mainstream solution due to its ability to process data streams with different sampling rates. Smyth was the first to propose an MRKF-based framework that integrates low-rate GNSS displacement data with high-rate acceleration data, enabling the accurate estimation of high-frequency displacement signals through a state-space model [32]. This method effectively utilizes the absolute positioning information from GNSS and the high-frequency dynamic information from accelerometers through two stages—prediction and update—thus laying a theoretical foundation for GNSS–accelerometer data fusion. However, the performance of conventional MRKF is highly sensitive to the accuracy of noise parameters. In practical engineering applications, the statistical properties of noise are often time-varying, non-Gaussian, and complex, making them difficult to model accurately using simple assumptions. Xu et al. proposed an enhanced MRKF that utilizes Maximum Likelihood Estimation (MLE) to optimize noise parameters, significantly improving GPS displacement accuracy and frequency bandwidth in long-span bridge monitoring [33]. However, its performance remains limited in the presence of outliers. To address this, Qu et al. developed a robust MRKF capable of real-time outlier detection and suppression, demonstrating that even sparse outliers can notably degrade modal frequency identification accuracy [34]. Further, to correct accelerometer-related errors such as bias and misalignment, they proposed a multi-antenna GNSS fusion method based on an unscented MRKF, achieving sub-millimeter displacement accuracy and enhancing overall fusion reliability in dynamic environments [35]. Their results demonstrate that multi-antenna GNSS systems can provide more accurate attitude information, offering a promising solution for accelerometer bias correction. Nevertheless, the practical application of such systems is still limited by high costs and complex installation requirements. Therefore, further research into low-cost correction methods is necessary.

**Figure 1:**
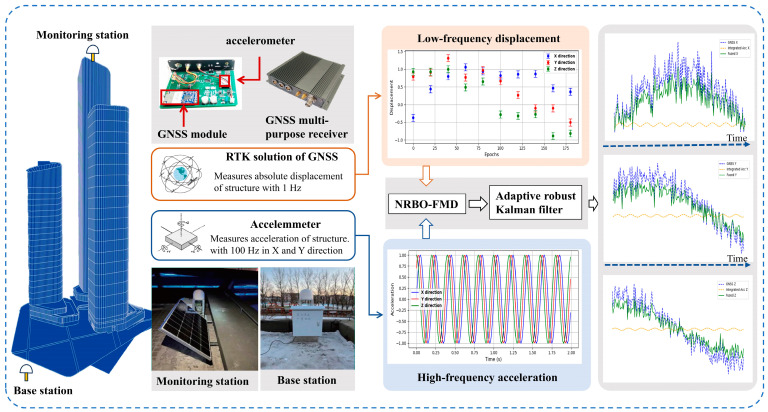
Review of dynamic deformation analysis of super-tall buildings based on GNSS and accelerometer data fusion based on NRBO-FMD adaptive anti-error Kalman filtering.

To meet the demand for real-time noise parameter estimation in the multi-rate fusion of GNSS and accelerometer data, this study introduces a fusion algorithm based on Newton–Raphson Optimization (NRBO), Feature Modal Decomposition (FMD), and Adaptive Robust Kalman Filtering (ARKF) [36]. The proposed NRBO-FMD-ARKF algorithm enables dynamic estimation of displacement noise parameters by integrating GNSS and accelerometer measurements, thereby significantly improving the accuracy and robustness of displacement estimation [37].To evaluate the performance of the algorithm, numerical simulations were conducted under various conditions, including different natural frequencies, randomly initialized noise parameters, and time-varying noise scenarios [38]. These simulations assessed the algorithm’s accuracy and stability in system displacement estimation. Subsequently, the 333 m-tall Changchun HaiRong Plaza was selected as a case study. Real-world monitoring data were employed to investigate the building’s dynamic response under actual operating conditions. Frequency-domain analyses were performed on the fused displacement results, raw accelerometer data, and GNSS RTK solutions. The results demonstrated that the proposed method offers clear advantages in identifying modal frequencies and provides a more comprehensive representation of the building’s dynamic characteristics. The main research process includes numerical validation, analysis of real-world operational data, and comparative frequency-domain analysis. The technical workflow of the proposed method is shown in Figure 1. This study presents an effective solution for the precise monitoring of dynamic displacement in super-tall buildings and offers new insights into the practical application of multi-sensor data fusion techniques.

## 2. GNSS and Accelerometer Data Fusion Method

### 2.1. NRBO-FMD Optimization Workflow

This section begins with a brief introduction to the traditional Newton–Raphson Optimization (NRBO) algorithm, followed by a detailed explanation of the Newton–Raphson Search Rule (NRSR) combined with the Trap-Avoidance Operator (TAO) [39]. Subsequently, the application of the NRBO algorithm in Feature Modal Decomposition (FMD) is discussed, with particular emphasis on the influence of filter size (FilterSize) and the number of modes (modenum) on decomposition performance. Based on optimization principles, a convergence criterion for NRBO in FMD is proposed, which effectively accelerates convergence and mitigates the risk of local optima, thereby improving the algorithm’s accuracy and stability in complex signal environments. In addition, an adaptive tuning factor is introduced by dynamically adjusting parameters during the iterative search process. This enhancement improves the algorithm’s noise sensitivity and facilitates rapid convergence to the optimal solution. Finally, the optimized algorithm is integrated with the traditional FMD approach [40], and the complete NRBO-FMD process is illustrated using a schematic diagram to demonstrate its advantages and applicability in non-stationary signal processing tasks.

#### 2.1.1. The Feature Modal Decomposition (FMD) Algorithm

The Feature Modal Decomposition (FMD) algorithm, an advancement over Empirical Mode Decomposition (EMD) and Ensemble Empirical Mode Decomposition (EEMD), places greater emphasis on signal decomposition accuracy and noise robustness [41]. FMD is a time–frequency-based technique specifically developed to extract physically meaningful Intrinsic Mode Functions (IMFs) from non-stationary, multi-component signals [42]. Its primary objective is to decompose complex signals into a series of intrinsic modes, thereby facilitating feature extraction and subsequent analysis. The algorithm achieves this by selecting and optimizing local characteristic scales while ensuring a zero local mean value. A key advantage of FMD lies in its ability to effectively mitigate noise interference in complex signal environments, resulting in improved decomposition accuracy. Consequently, FMD has been widely applied in signal feature extraction, time–frequency analysis, and dynamic monitoring, demonstrating strong potential across various research and engineering domains [43].

The goal of Feature Mode Decomposition (FMD) is to decompose a non-stationary, multi-component signal *x*(*t*) into several Intrinsic Mode Functions *IMF_i_*(*t*), and a residual signal *r*(*t*). The signal decomposition formula can be expressed as follows:

(1) $$x\left(t\right)=\sum_{i=1}^{n}{IMF}_{i}(i)+r(t)$$

As a decomposition method suitable for nonlinear and non-stationary signals, the process begins with the input signal *x*(*t*). By identifying the local extrema of the signal, the component range of the intrinsic modal features is determined. All local maxima and minima of the signal are extracted, and spline interpolation is used to generate the upper envelope $u_{upper}(t)$ and lower envelope $u_{lower}(t)$. The mean of these envelopes is then calculated:

(2) $$m\left(t\right)=\frac{u_{upper}\left(t\right)+u_{lower}(t)}{2}$$

By subtracting the local mean value *m*(*t*) from the signal *x*(*t*), the candidate for the current modal component is computed as follows:

(3) $$h\left(t\right)=x(t)-m\left(t\right)$$

If *h*(*t*) satisfies the conditions for an intrinsic mode function (IMF), it is recorded as the first modal component, IMF_1_. Otherwise, *h*(*t*) undergoes iterative sifting until the conditions are met. Once the current modal component is extracted, the residual signal is calculated as follows:

(4) $$r\left(t\right)=x(t)-{IMF}_{1}$$

The residual signal *r(t)* is used as the input for the next iteration, and the above procedure is repeated until the residual becomes monotonic or satisfies predefined termination criteria. This iterative process yields a set of extracted modal components {IMF_1_, IMF_2_, …, IMF_n_} along with the final residual signal. The decomposition process takes into account both the periodicity of the data signal and the characteristics of structural deformation, while maintaining robustness against external interference and noise. By employing an adaptive finite impulse response (FIR) filter to extract decomposition modes, the method eliminates dependence on predefined filter shape, bandwidth, or center frequency, thus enabling more comprehensive signal decomposition. Moreover, FMD does not require prior knowledge of periodic deformation patterns and can effectively isolate complex signal components dominated by specific frequency bands within the monitoring data, thereby significantly enhancing decomposition accuracy [44]. In addition to suppressing noise interference, the method also yields modal components with clear physical significance, improving the interpretability and reliability of the analysis results [22].

#### 2.1.2. Newton–Raphson Optimization Algorithm (NRBO)

The Newton–Raphson-Based Optimizer (NRBO) is a metaheuristic algorithm that integrates the rapid convergence properties of the Newton–Raphson method with the global search capabilities of metaheuristic techniques [45]. To enhance its overall performance, NRBO incorporates two key mechanisms: the Newton–Raphson Search Rule (NRSR) and the Trap-Avoidance Operator (TAO). The NRSR leverages the Newton–Raphson method to improve local search performance and convergence speed, enabling efficient identification of optimal solutions in complex search spaces. In contrast, the TAO is designed to prevent entrapment in local optima by guiding the search process away from suboptimal regions, thereby enhancing global exploration ability [46]. This dual-structured approach enables NRBO to effectively balance exploration and exploitation, making it well suited for solving challenging optimization problems across diverse and complex landscapes.

The Newton–Raphson Search Rule (NRSR) is a core component of the Newton–Raphson Optimization Algorithm (NRBO), designed to approximate solutions to nonlinear equations in both real and complex domains. By utilizing the Taylor series expansion, NRSR dynamically adjusts the search direction and step size based on the second-order derivative information of the objective function, thereby improving both convergence speed and accuracy. The position update formula is expressed as follows:

(5) $$x_{n+1}=x_{n}-\frac{f^{\prime }\left(x_{n}\right)}{f^{\prime \prime }\left(x_{n}\right)},\;n=1,2,3,\cdots$$

The NRSR control vector enables more precise exploration of the feasible region and facilitates the identification of superior positions. The second derivative is expressed as follows:

(6) $$f\left(x+∆x\right)=f\left(x\right)+f^{\prime }\left(x_{0}\right)∆x+\frac{1}{2!}f^{\prime \prime }\left(x_{0}\right)∆x^{2}+\frac{1}{3!}f^{\prime \prime }\left(x_{0}\right)∆x^{3}+\cdots$$

(7) $$f\left(x-∆x\right)=f\left(x\right)-f^{\prime }\left(x_{0}\right)∆x+\frac{1}{2!}f^{\prime \prime }\left(x_{0}\right)∆x^{2}-\frac{1}{3!}f^{\prime \prime }\left(x_{0}\right)∆x^{3}+\cdots$$

The expressions for *f*′(*x*) and *f*″(*x*) are derived as follows:

(8) $$f^{\prime }\left(x\right)=\frac{f\left(x+∆x\right)-f\left(x-∆x\right)}{2∆x}$$

(9) $$f^{\prime \prime }\left(x\right)=\frac{f\left(x+∆x\right)+f\left(x-∆x\right)-2\times f\left(x\right)}{∆x^{2}}$$

The position update formula is as follows:

(10) $$x_{n+1}=x_{n}-\frac{(f\left(x_{n}+∆x\right)-f\left(x_{n}-∆x\right))\times \Delta x}{2\times \left(f\left(x_{n}+∆x\right)-f\left(x_{n}-∆x\right)-2\times f(x_{n})\right)}$$

The NRSR is a key component of the NRBO. Considering a population-based search approach, partial modifications are proposed as follows:

(11) $$NRSR=randn\times \frac{\left(X_{\omega }-X_{b}\right)\times \Delta x}{2\times \left(X_{\omega }+X_{b}-2\times x_{n}\right)}$$

Here, *X_w_* represents the worst position, and *X_b_* denotes the best position. The adaptive coefficient *δ* balances the exploration and exploitation capabilities.

(12) $$\delta ={\left(1-\left(\frac{2\times IT}{{Max}_{1}T}\right)\right)}^{5}$$

The expression of Δ*x* is as follows:

(13) $$\Delta x=rand(1,dim)\times \left|X_{b}-X_{n}^{IT}\right|$$

*X_b_* represents the current optimal solution, and the NRSR is expressed as follows:

(14) $$x_{n+1}=x_{n}-NRSR$$

The parameter ρ is introduced to enhance the exploitation capability of the proposed NRBO, guiding the population in the correct direction.

(15) $$x_{n+1}=x_{n}-NRSR$$

### 2.2. Acceleration Frequency-Domain Integral

When integrating acceleration in the time domain, a zero-drift phenomenon occurs, leading to the gradual accumulation of errors and causing the calculated displacement to deviate from the true value [47]. By performing integration in the frequency domain, displacement data can be obtained more quickly and stably. Unlike time-domain integration, frequency-domain integration first requires a Fourier transform. Using the Fourier transform, the accelerometer data are processed in the frequency domain, followed by an inverse Fourier transform to obtain velocity and displacement information in the time domain. The fundamental principles are illustrated in the diagram below.

**Figure 2:**
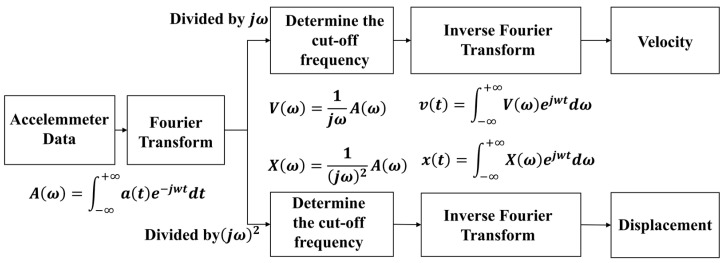
Frequency-domain integration calculation process.

In the Figure 2, F represents the Fourier transform, *a*(*t*) denotes the acceleration signal, and *ω* corresponds to the frequency components in the Fourier domain. By applying frequency-domain integration, the acceleration signal can be effectively converted into velocity and displacement signals.

### 2.3. Adaptive Robust Kalman Filter (ARKF)

Adaptive Robust Kalman Filtering is an improved Kalman filtering method designed to address scenarios where observation noise exhibits a strongly non-Gaussian distribution (e.g., containing outliers) or where the statistical characteristics of the noise are unknown [48]. The core concept involves incorporating robust weighting and adaptive adjustment strategies within the traditional Kalman filtering framework. In this study, dynamic adjustments to both measurement noise and process noise are implemented to further enhance robustness.

**Figure 3:**
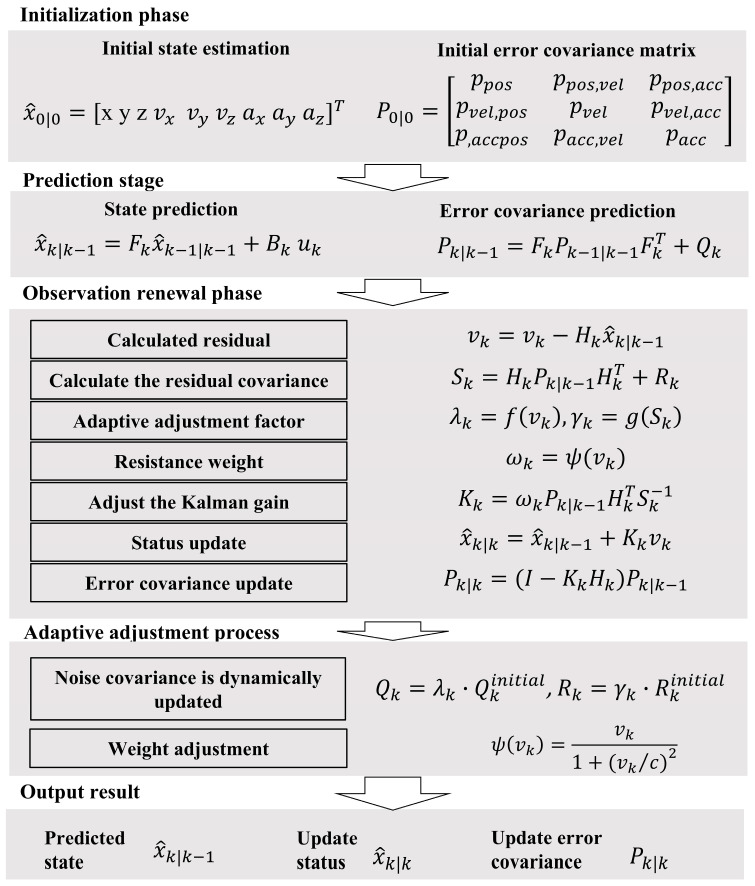
Adaptive Robust Kalman Filter flow chart.

In the Adaptive Robust Kalman Filter (Figure 3), the state vector *x_k_* represents the dynamic state of the system, while the observation vector *z_k_* corresponds to measurements of the state. *F_k_* denotes the state transition matrix, *B_k_* represents the control input matrix, and *u_k_* is the control input. The observation matrix *H_k_* maps the state to the observation space. The robust weighting factor *w_k_* is introduced to mitigate the influence of outliers in the measurements. The observation residual, defined as $\nu_{k}=z_{k}-H_{k}{\hat{x}}_{k|k-1}$, quantifies the discrepancy between the predicted and observed values.

The noise covariance matrices *Q_k_* and *R_k_* characterize the dynamic properties of process noise and measurement noise, respectively. Adaptive factors λ_k_ lambda_k and γ_k_ gamma_k are employed to update these noise characteristics dynamically. The robust function ψ(⋅) constrains the impact of outliers on the filtering process. ${\hat{x}}_{k|k-1}$ represents the predicted state, while ${\hat{x}}_{k|k}$ denotes the updated state. *P_k_*_∣_*_k−_*_1_ and *P_k_*_∣_*_k_* are the predicted and updated error covariance matrices, respectively. The adjusted Kalman gain *K_k_* is computed to optimize the state update by balancing the contributions of the predicted state and the observed data. This framework ensures robustness and adaptability in dynamic and noisy environments, making it particularly effective for applications with non-Gaussian or time-varying noise characteristics.

### 2.4. Overall Dynamic Displacement Reconstruction Method

**Figure 4:**
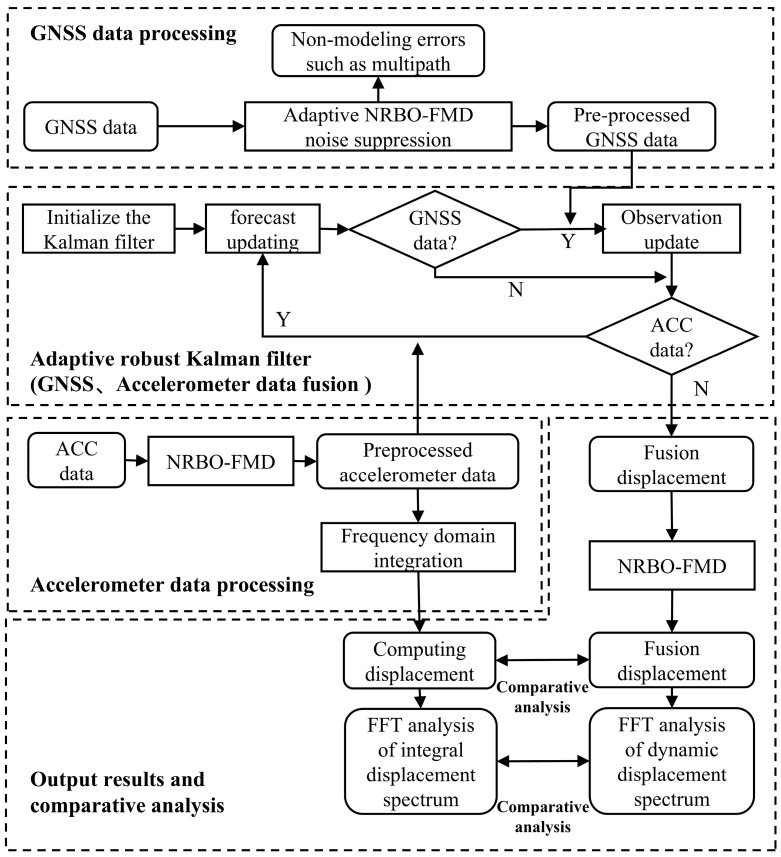
Diagram of the integrated GNSS/accelerometer dynamic deformation analysis process.

The integrated dynamic displacement reconstruction technique leverages the complementary advantages of Global Navigation Satellite System (GNSS) and accelerometer (ACC) sensors [49]. As shown in Figure 4, First, the NRBO-FMD algorithm is applied to GNSS data to mitigate unmodeled errors, such as multipath effects, yielding preprocessed GNSS displacement. Simultaneously, ACC data undergo noise suppression via NRBO-FMD, followed by frequency-domain integration to obtain displacement estimates. Subsequently, the preprocessed GNSS and ACC displacements are fused using an Adaptive Robust Kalman Filter (ARKF), which incorporates both prediction and observation update mechanisms to reconstruct high-accuracy dynamic displacement. The reconstructed displacement is then compared with the ACC-derived displacement obtained through frequency-domain integration. Finally, Fast Fourier Transform (FFT) is applied to analyze the dynamic displacement spectrum from the fused data and the integrated displacement spectrum from ACC data. This approach combines multi-sensor data fusion with spectral comparison analysis, offering a robust and reliable framework for dynamic deformation monitoring.

## 3. Assessment Indicators and Numerical Simulation

In this study, three evaluation metrics were employed to assess the estimation error, prior estimation accuracy, and posterior estimation accuracy of the proposed NRBO-FMD-ARKF algorithm. To enable a comprehensive comparison with conventional denoising and data fusion methods, a hybrid cosine function was constructed as the reference signal. Numerical simulation results were then used to validate the superiority of the proposed algorithm in terms of noise suppression and multi-source data fusion performance, as well as its effectiveness and adaptability under varying operational conditions. This comprehensive evaluation highlights the robustness and precision of the NRBO-FMD-ARKF algorithm, demonstrating its strong potential for advanced applications in dynamic system monitoring and integrated sensor data processing.

### 3.1. Evaluation Indicators

In this study, the root mean square error (RMSE) is used to quantify the discrepancy between the estimated and actual deformations [50,51]. Additionally, the posterior estimation standard deviation (POSD) is employed to assess the algorithm’s performance in terms of both prior and posterior estimation accuracy [52]. The specific definitions of these two accuracy evaluation metrics are provided below.

(1) Root Mean Square Error (RMSE):

This represents the overall error between the estimated deformation and the true deformation, reflecting the algorithm’s precision in capturing dynamic characteristics. A smaller RMSE indicates lower estimation error and higher overall accuracy, which is defined as follows:

(16) $$RMSE=\sqrt{\frac{1}{n}\sum_{1=1}^{n}{\left({\hat{x}}_{i}-x_{i}\right)}^{2}}$$

Here, ${\hat{x}}_{i}$ represents the displacement estimated by the proposed algorithm, $x_{i}$ denotes the true displacement, and n is the number of samples.

(2) Prior Estimation Standard Deviation (PESD):

This measures the algorithm’s capability to provide accurate prior estimates before incorporating real-time data. PESD indicates that the model’s prior estimation is more reliable, which is defined as follows:

(17) $$PESD=\sqrt{\frac{1}{n}\sum_{1=1}^{n}{\left(\sigma_{prior,i}\right)}^{2}}$$

Here, $\sigma_{prior}$ represents the standard deviation of the prior estimation error at the i-th time point.

(3) Posterior Estimation Standard Deviation (POSD):

This assesses the precision of the algorithm’s estimates after integrating observation data, highlighting its effectiveness in reducing uncertainties. A smaller POSD indicates higher accuracy of the posterior estimation results, highlighting the effectiveness of the algorithm, which is defined as follows:

(18) $$POSD=\sqrt{\frac{1}{n}\sum_{1=1}^{n}{\left(\sigma_{post,i}\right)}^{2}}$$

Here, $\sigma_{post}$ represents the standard deviation of the posterior estimation error at the *i*-th time point.

### 3.2. Numerical Simulation

To comprehensively evaluate the performance of the proposed NRBO-FMD-ARKF algorithm, two signal scenarios with varying noise levels were simulated, The parameters are shown in Table 1. Considering the deformation characteristics of super-tall buildings and the sensing properties of GNSS and accelerometers, a hybrid cosine signal simulation scheme was developed. This scheme models both the quasi-static and dynamic deformation behaviors of super-tall buildings under wind-induced vibrations and complex loading conditions.

(19) $$x\left(t\right)=bcos\left(2\pi f_{1}t\right)+hcos(2\pi f_{2}t)$$

Here, *b* and *h* represent amplitude constants (unit: mm), while *f*_1_ and *f*_2_ correspond to the primary frequency components (unit: Hz). The corresponding acceleration signal is derived as the second-order derivative of the displacement signal.

(20) $$a\left(t\right)=\frac{d^{2}x(t)}{dt^{2}}$$

The GNSS signal is sampled at a frequency of 1 Hz, primarily capturing low-frequency quasi-static displacement components. White noise $n_{z}\sim N(0,\sigma_{x}^{2})$ is added to simulate sensor noise, resulting in the final GNSS measurement signal:

(21) $$z_{GNSS}\left(t\right)=x\left(t\right)+n_{z}$$

The acceleration signal is sampled at a frequency of 100 Hz, capturing high-frequency dynamic deformation. White noise $n_{a}\sim N(0,\sigma_{a}^{2})$ and bias drift error *δ* are added to the signal, resulting in the following measurement signal:

(22) $$z_{acc}\left(t\right)=a\left(t\right)+n_{a}\;$$

### 3.3. Performance Analysis

(1) Validation of the Effectiveness of the NRBO-FMD Algorithm in Mitigating GNSS Errors

**Figure 5:**
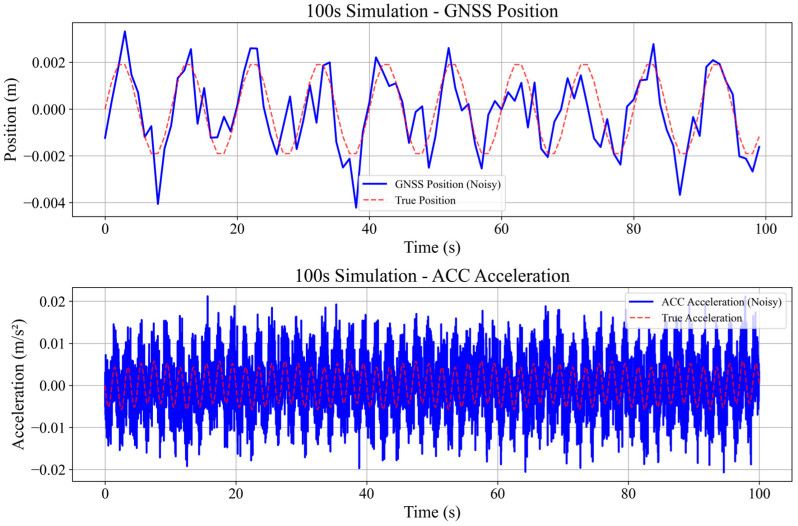
Simulation and generation of 100-s GNSS displacement and accelerometer plots to illustrate the dynamic structural response of a building over a short time period.

**Figure 6:**
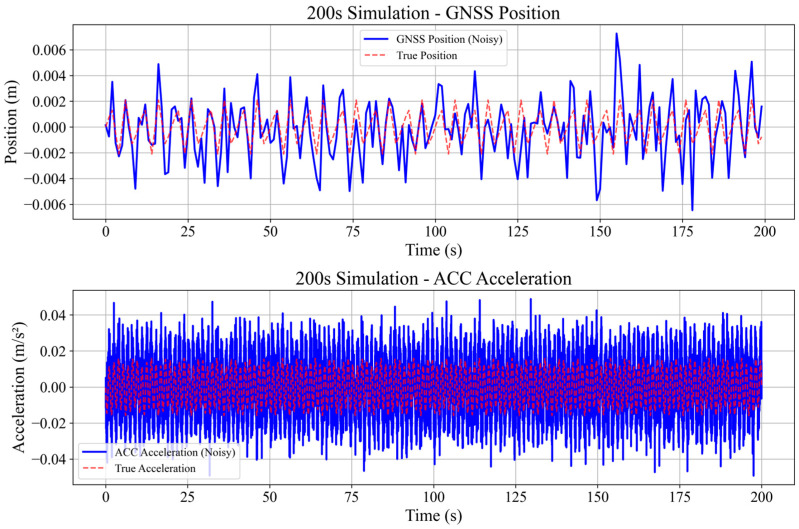
Simulation and generation of 200 s GNSS displacement and acceleration plots to model the routine dynamic behavior of the building.

The numerical simulation results are presented in Figure 5 and Figure 6. GNSS deformation data were generated using a hybrid cosine signal, ensuring that the simulated noise reflects the quasi-periodic characteristics of GNSS multipath errors. To accurately model the complex fluctuations of these errors, a combination of sinusoidal waves with varying frequencies—both low and high—was employed. This method effectively captures the intricate variation patterns of multipath interference. The NRBO-FMD algorithm was applied to the noise-contaminated simulation data for denoising. The performance of the denoising process was evaluated using three metrics, root mean square error (RMSE), signal-to-noise ratio (SNR), and Correlation Coefficient (R). The results demonstrate that the NRBO-FMD algorithm significantly reduces RMSE and improves SNR. For the 100 s dataset, the NRBO-FMD algorithm achieved an RMSE of 0.0007, notably lower than those obtained by wavelet denoising, Kalman filtering, and the Empirical Mode Decomposition (EMD) method. Additionally, it improved the SNR by 3.0186 dB, exceeding the enhancement levels of the comparison methods. Similarly, for the 200 s dataset, the NRBO-FMD algorithm maintained superior performance, achieving an RMSE of 0.0010 and an SNR improvement of 5.9980 dB, again outperforming other denoising techniques. Overall, the NRBO-FMD method demonstrated excellent accuracy and SNR enhancement, confirming its effectiveness in noise reduction and its reliability for subsequent sensor data fusion analysis.

(2) Validation of the Effectiveness of the NRBO-FMD Algorithm in Removing High-Frequency Noise from ACC Data

**Figure 7:**
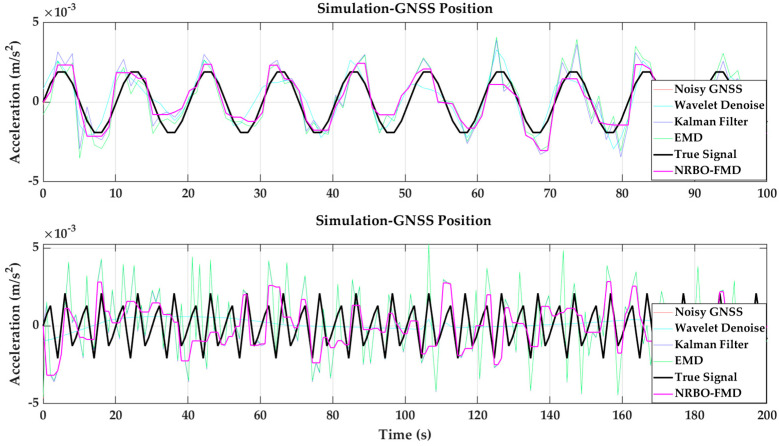
Validation of the effectiveness of the NRBO-FMD algorithm in mitigating GNSS hybrid errors and comparison with various denoising algorithms.

**Figure 8:**
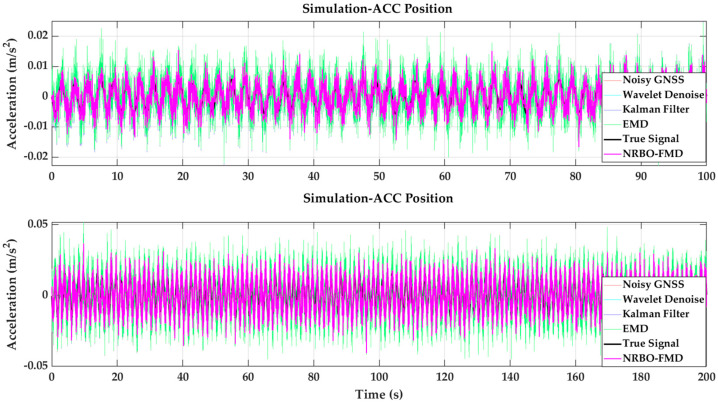
Validation of the effectiveness of the NRBO-FMD algorithm in high-frequency noise suppression for ACC data and comparison with various denoising algorithms.

Accelerometers are highly susceptible to high-frequency noise during real-world measurements. In the simulation dataset, this noise exhibits pronounced high-frequency characteristics, typically originating from sensor vibrations and environmental disturbances, resulting in strong random fluctuations. To accurately replicate the high-frequency noise behavior of accelerometer (ACC) sensors, a synthetic dataset was constructed by superimposing multiple high-frequency sinusoidal signals and introducing random noise. This approach provides a realistic representation of measurement errors in complex environments, as illustrated in Figure 7 and Figure 8. Experimental results demonstrate that the NRBO-FMD algorithm offers significant advantages in suppressing high-frequency noise in ACC data. For the 100 s dataset, the RMSE of the denoised signal using NRBO-FMD was 0.0030, which is lower than that achieved by Kalman filtering (0.0050) and the Empirical Mode Decomposition (EMD) method (0.0050), underscoring its superior noise reduction capability. Similarly, for the 200 s dataset, NRBO-FMD maintained effective denoising performance, achieving an RMSE of 0.0062 and an SNR improvement of 4.1488 dB, significantly outperforming Kalman filtering (0.0849 dB) and the EMD method (–0.0035 dB). Overall, the NRBO-FMD algorithm demonstrates strong capabilities in attenuating high-frequency noise in ACC data, particularly in mitigating random high-frequency interference. These results confirm the algorithm’s enhanced stability and robustness in noise suppression, validating its suitability for accelerometer noise reduction applications.

(3) Validation of the Effectiveness of the NRBO-FMD-ARKF Algorithm in GNSS and ACC Data Fusion

**Figure 9:**
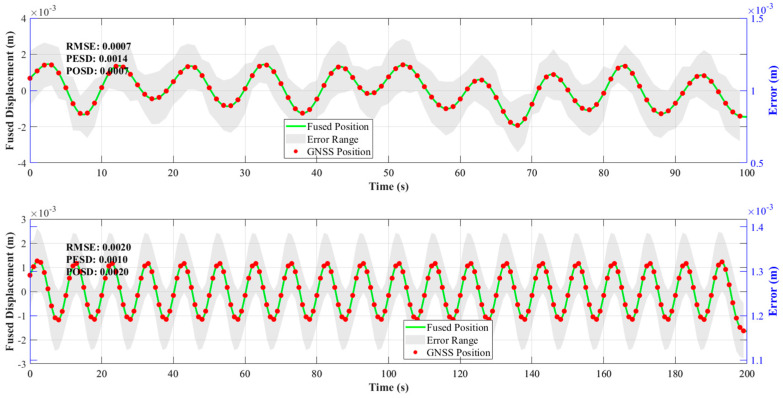
Validation of the effectiveness of the NRBO-FMD-ARKF algorithm in GNSS and ACC data fusion.

As shown in Figure 9, the displacement variations obtained through GNSS and ACC data fusion are presented for both the 100 s and 200 s datasets. In Figure 9, red dots represent GNSS observations, the green curve denotes the fused displacement data, and the gray-shaded area indicates the error range. The NRBO-FMD-ARKF algorithm effectively integrates the low-frequency characteristics of GNSS data with the high-frequency information from ACC sensors, achieving a complementary fusion. This fusion process successfully mitigates GNSS multipath errors while significantly reducing high-frequency noise in ACC measurements, resulting in smoother and more stable fused displacement data. From an error evaluation perspective, the 100 s dataset achieved an RMSE of 0.0007, with both PESD and POSD at 0.0010. For the 200 s dataset, the RMSE was 0.0019, with PESD and POSD also at 0.0019. These results indicate that the fused data maintain high accuracy over extended observation periods. Specifically, for the 100 s dataset, the fused data closely fit the GNSS observation points while effectively suppressing irregular error fluctuations. In the 200 s dataset, where the high-frequency characteristics of the ACC data are more pronounced, the NRBO-FMD-ARKF algorithm successfully suppresses high-frequency noise, maintaining a low error range and ensuring data stability and reliability. Overall, the NRBO-FMD-ARKF algorithm fully leverages the complementary strengths of GNSS and ACC data, resulting in fused measurements that not only minimize error influence but also significantly enhance measurement accuracy and robustness against interference. These findings validate the effectiveness of the proposed method in high-precision displacement monitoring and multi-sensor data fusion, offering strong support for real-world applications.

## 4. Experimental Analysis

**Figure 10:**
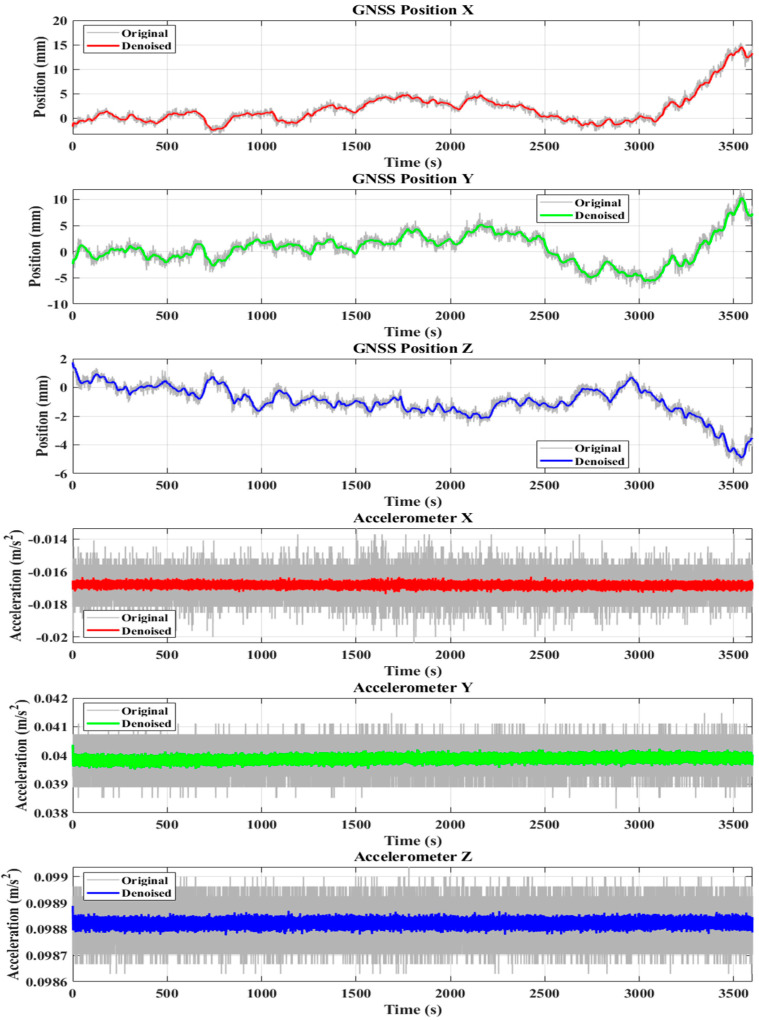

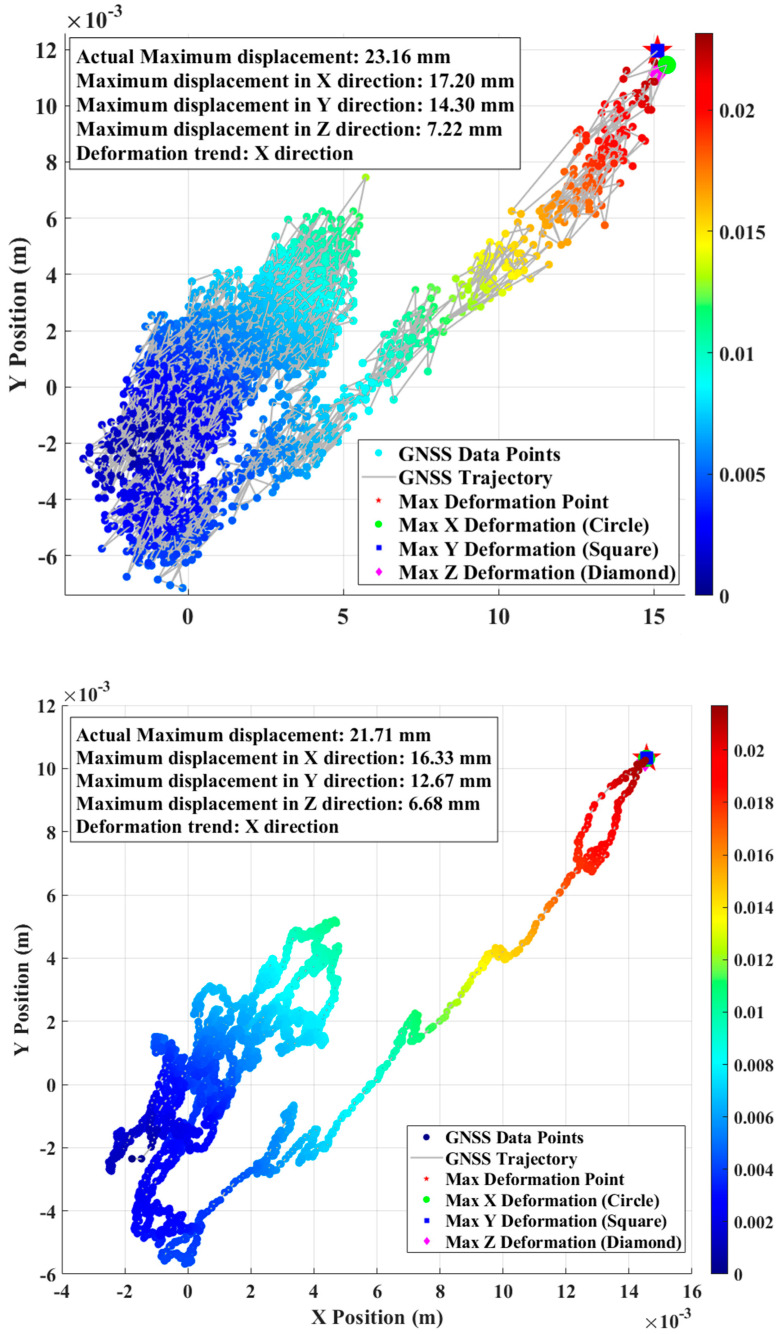
Demonstration of the NRBO-FMD algorithm’s noise reduction effect, which shows the original data after noise reduction, compares the three-dimensional changes in the deformation displacement trajectory before and after noise reduction, and highlights the maximum displacement points extracted in each direction.

In this study, deformation monitoring data collected on 28 October 2022, from the Hairong Plaza Building in Changchun, were analyzed. The building is approximately 200 m tall. An integrated device combining a satellite receiver and an accelerometer was employed for data acquisition (see Table 2 for specific parameters). The installation site of the monitoring equipment is shown in Figure 1. The reference station was installed on the rooftop of a nearby stable building, specifically on the second floor in an open area, while the monitoring station was deployed on-site to conduct measurements. The GNSS receiver operated at a sampling frequency of 1 Hz, while the accelerometer sampled at 100 Hz. Both instruments recorded structural deformation data, and the GNSS and accelerometer time series used in this analysis are presented in Figure 10. This study focuses on three-dimensional deformation in coordinate directions, using one hour of monitoring data from 28 October 2022 for analysis. The NRBO-FMD algorithm was applied to the raw GNSS data to remove multipath effects, unmodeled errors, and certain high-frequency noise components, thus completing the preprocessing of the GNSS data. Similarly, the raw accelerometer data were preprocessed to eliminate low-frequency trends and minor noise artifacts.

In the analysis of deformation data for super-tall buildings, a standardized coordinate system was established to unify the GNSS and accelerometer data within a consistent reference frame. The building’s structural coordinate system was adopted for this purpose. In this system, the X-axis aligns with the building’s longitudinal direction, the Y-axis with its transverse direction, and the Z-axis points vertically downward, following the direction of gravity. Initially, the GNSS observation data, originally recorded in the WGS84 geodetic coordinate system, were transformed and corrected for orientation to align with the structural coordinate system of the building. During installation, the accelerometer axes were carefully aligned with the building’s coordinate system, and orientation consistency was ensured through the use of an attitude sensor or an initial calibration procedure. This alignment enabled both the GNSS displacement data and the accelerometer data to be fused along the same XYZ axes, thereby enhancing the accuracy and comparability of the deformation monitoring results.

As shown in Figure 10, the NRBO-FMD algorithm demonstrated excellent noise reduction performance and exhibited strong adaptability to various noise interference scenarios, including GNSS multipath errors and high-frequency noise in accelerometer data. The algorithm significantly reduced noise in both GNSS position components (X, Y, Z) and accelerometer readings (ax, ay, az), resulting in smoother signals and improved data interpretability. Furthermore, the GNSS deformation trajectory clearly shows that the NRBO-FMD-processed data reveal a more stable deformation trend, with clearer displacement patterns and more accurate directional indications. The algorithm effectively suppresses both high- and low-frequency noise while preserving essential structural response features. As a result, it provides reliable data support for subsequent structural health monitoring and safety assessment tasks.

**Figure 11:**
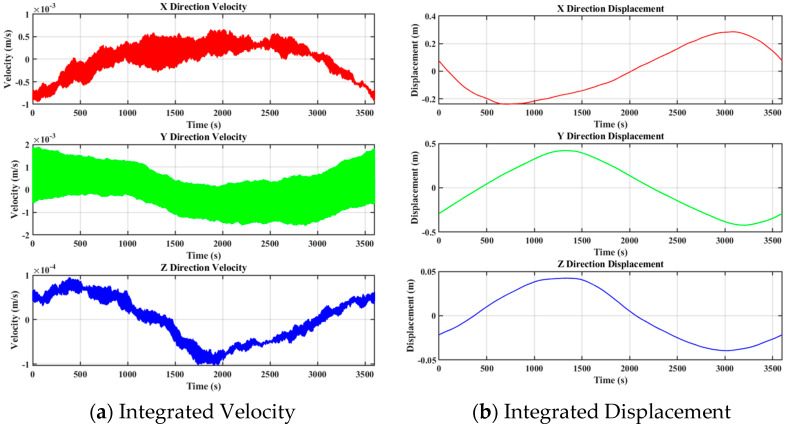
Accelerometer Integration Results: (**a**) Velocity Time History Obtained from Data Integration; (**b**) Displacement Time History Obtained from Velocity Integration.

After noise removal from the accelerometer data, the velocity time history (Figure 11a) reveals the building’s vibration velocity characteristics in three directions. As shown in Figure 11, the velocity fluctuation amplitudes in the X and Y directions are approximately 0.005 m/s, while changes in the Z direction (vertical) are relatively minimal. The velocity data directly reflect the building’s dynamic response to external wind loads.

The displacement time history (Figure 11b) is obtained by integrating the velocity data in the time domain, illustrating the displacement variation of the building in all three directions. Due to accelerometer zero drift, the accumulated integration error causes a deviation between the calculated displacement and the true value. The maximum displacement in the X direction is approximately 0.2 m, while the displacement in the Y direction ranges from −0.5 to 0.5 m. The displacement in the Z direction remains relatively small, fluctuating within ±0.05 m, which significantly differs from the displacement measured by GNSS. Consequently, accelerometer data cannot be directly used for quantitative monitoring of building deformation. However, given the high sampling frequency and low monitoring cost of the accelerometer, it can effectively capture high-frequency dynamic deformations in super-tall buildings, providing valuable qualitative information on whether the building structure remains within a safe range.

**Figure 12:**
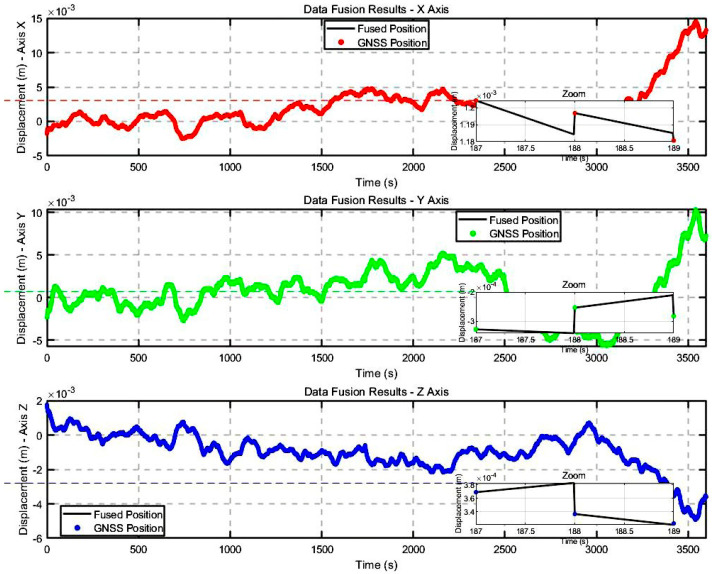
Triaxial deformation direction data fusion results.

In Figure 12, a comparison is presented between the GNSS displacement time series and the accelerometer data after denoising, alongside the displacement time series obtained via Adaptive Robust Kalman Filtering. The fused data integrate 1 Hz GNSS data with accelerometer measurements, correcting errors accumulated during accelerometer integration by adjusting each GNSS epoch. This approach significantly reduces error accumulation from accelerometer integration and compensates for the limitations of high-frequency GNSS hardware, such as its low sampling frequency and high cost.

The proposed method overcomes the limitations of individual sensors and provides more accurate displacement measurements. The zoomed-in window in Figure 12 clearly illustrates the high precision of the fused data, which is critical for real-time structural health monitoring. Compared to the original GNSS and accelerometer measurements( As shown in Table 3), the fused displacement data demonstrate significant improvements in both accuracy and stability. Specifically, the root mean square error (RMSE) decreased from 0.00515 m to 0.00362 m after fusion, indicating a substantial enhancement in the overall fit to true displacement. Similarly, the Position Error Standard Deviation (PESD) decreased from 0.00428 m to 0.00257 m, and the Position Offset Standard Deviation (POSD) was reduced from 0.00673 m to 0.00476 m, reflecting a notable reduction in error dispersion and offset across all axes. These improvements highlight the effectiveness of the fusion method in mitigating sensor-specific noise and drift. Furthermore, the fused time series exhibits no significant drift or abrupt fluctuations, demonstrating excellent consistency and temporal stability. Overall, these metrics confirm that the proposed fusion algorithm not only enhances measurement precision but also ensures reliable performance over extended monitoring periods, making it highly suitable for long-term structural health monitoring applications.

The high-dynamic monitoring system, which integrates GNSS and accelerometer data fusion, provides robust technical support for the safety management of super-tall buildings. Through the algorithmic processing workflow proposed in this paper, the system enhances the data sampling frequency while effectively filtering out redundant noise. This integration leverages the complementary advantages of both sensors, allowing for the accurate extraction of dynamic deformation information and improving the precision of dynamic deformation monitoring. This approach not only facilitates real-time monitoring of a building’s response to environmental loads but also supports the validation of structural design by comparing the observed data with the expected design values.

Based on the empirical formula for estimating the fundamental natural period of high-rise building structures—typically ranging from [0.05–0.1]·*N* (where *N* is the number of above-ground floors)—and according to the Nyquist theorem, 1 Hz GNSS deformation data can effectively capture building vibration characteristics in the 0–0.5 Hz frequency range. For the building under study, which has 67 floors above ground, the estimated fundamental natural period is approximately 3.35–6.7 s, corresponding to a primary modal frequency range of 0.149–0.298 Hz.

**Figure 13:**
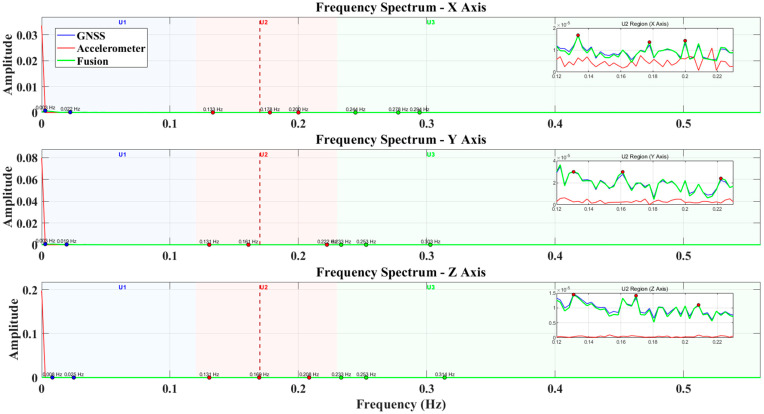
The power spectral density of accelerometer data (red), GNSS data (green), and fused displacement (blue) was extracted along the XYZ three-axis directions.

Based on the building’s frequency response characteristics and signal sources, the spectrum in the experiment (as shown in Figure 13) is divided into three typical regions: (1) U1 (Static/Quasi-static Response Zone): Frequency range of 0–0.12 Hz. This region mainly reflects the building’s slow deformation response to gradually varying loads such as wind and ambient temperature. GNSS contributes significantly in this zone, exhibiting strong low-frequency displacement detection capabilities. (2) U2 (Mid-to-Low-Frequency Disturbance Zone): Frequency range of 0.12–0.23 Hz. This region is primarily influenced by wind loads, thermal expansion and contraction, and GNSS multipath effects. A distinct spectral peak is often observed around 0.17 Hz. In this zone, both GNSS and fused GNSS–accelerometer data effectively identify subtle structural disturbances. (3) U3 (Primary Structural Mode Response Zone): Frequency range of 0.149–0.298 Hz. This corresponds to the expected range of the building’s fundamental vibration modes, capturing its main structural responses under external excitations such as earthquakes or strong winds. In this zone, the fused spectrum shows a noticeable energy concentration, marking it as a critical frequency band for structural health monitoring.

The monitoring in this study covers a spectral range of 0.003–0.314 Hz, fully encompassing the U1–U3 frequency zones. Observations of the X/Y/Z-axis spectra indicate a clear peak near 0.17 Hz in the U2 zone and increased energy in the U3 zone, suggesting stable structural response characteristics. These results confirm the effectiveness and sensitivity of the data fusion algorithm in identifying structural features across different frequency bands.

## 5. Conclusions

To accurately capture the dynamic displacement of super-tall buildings under complex working conditions, this study proposes a data fusion algorithm based on NRBO-FMD optimization and Adaptive Robust Kalman Filtering (ARKF). The algorithm first utilizes NRBO-FMD to preprocess GNSS and accelerometer data, effectively mitigating GNSS multipath effects and other unmodeled errors, as well as high-frequency noise from the accelerometer. Subsequently, the preprocessed GNSS and accelerometer data are fused using ARKF, achieving high-precision dynamic displacement reconstruction.

Numerical simulations were conducted under different conditions to assess the algorithm’s accuracy in estimating displacement at varying noise levels. Additionally, using this algorithm, GNSS and accelerometer data collected from the field were fused to estimate the three-dimensional dynamic displacement of the Hairong Square Building in Changchun under real-world conditions. The main findings are summarized as follows:

1. The NRBO-FMD algorithm demonstrated excellent performance in suppressing noise from both GNSS and accelerometer data. For GNSS data, the RMSE of the 100 s dataset decreased to 0.0007, and the SNR improved by 3.0186 dB. For the 200 s dataset, the RMSE reduced to 0.0010, with a 5.9980 dB improvement in SNR. For accelerometer data, the RMSE of the 100 s dataset dropped to 0.0030, and for the 200 s dataset, it decreased to 0.0062, with a 4.1488 dB increase in SNR. These results indicate that the NRBO-FMD algorithm effectively processes various types of noise while retaining critical information.
2. The NRBO-FMD-ARKF fusion algorithm showed excellent performance in displacement estimation. For the 100 s dataset, the RMSE was 0.0007, with both PESD and POSD values of 0.0010. For the 200 s dataset, the RMSE, PESD, and POSD were all 0.0019. These results show that the fused data maintain high accuracy over long observation periods and effectively suppress irregular error fluctuations.
3. In practical applications, the algorithm successfully fused 1 Hz GNSS data and 100 Hz accelerometer data, overcoming the limitations of a single sensor and providing more precise displacement measurements. The fusion results showed an RMSE of 0.003618 m, with PESD of 0.00257 m and POSD of 0.00476 m, demonstrating good performance in both accuracy and stability. Spectral analysis results showed that the fused displacement data more comprehensively reflected the building’s dynamic response characteristics, including multiple major frequency components ranging from 0.003 Hz to 0.314 Hz. This assists in identifying the building’s natural frequency, tracking structural stiffness changes, and detecting potential structural performance degradation early.

The method proposed in this study provides an effective solution for long-term displacement monitoring of super-tall buildings in complex environments, expanding the application of GNSS–accelerometer combinations in structural health monitoring. Future research directions include the development of real-time and even predictive full-scale dynamic displacement estimation methods to further improve the timeliness and early-warning capabilities of monitoring systems.

## Figures and Tables

**Table 1:** Simulation parameter table.

Dataset	Amplitude (mm)	Frequency (Hz)	$\mathrm{Noise}\;(\sigma_{a},$σ)	Simulation Duration (s)
1	b = 20, h = 5	f1 = 0.1, f2 = 0.5	0.005 m/s^2^, 0.001 m	100
2	b = 15, h = 7	f1 = 0.2, f2 = 0.07	0.02 m/s^2^, 0.01 m	200

**Table 2:** Principal specifications of GNSS and accelerometer.

Equipment	Performance	
GNSS	Signal tracking	BDS: B1/B2/B3; GPS: L1/L2/L5; GLONASS: L1/L2; GALILEO: E1/E5a/E5b; QZSS: L1/L5;
	RTK(RMS)	Horizontal: ±8 mm + 1 ppm; Vertical: ±15 mm + 1 ppm
	Updating frequency	1 Hz
Accelerometer	Measurement range	6 g
	Noise density	37 μg/$\sqrt{Hz}$
	Offset error	1.15 mg
	Linearity error	1 mg
	Initial bias error (one year)	10 mg

**Table 3:** Comparison of noise characteristics before and after fusion using the proposed method.

Metric	GNSS Displacement/Accelerometer (Before)	Fused Displacement (After)
RMSE (m)	0.00515	0.00362
PESD (m)	0.00428	0.00257
POSD (m)	0.00673	0.00476

## Data Availability

Data are only available upon request due to restrictions regarding, e.g., privacy and ethics. The data presented in this study are available from the corresponding author upon request. The data are not publicly available due to their relation to other ongoing research.
